# Inhibited inositol monophosphatase and decreased myo‐inositol concentration improve wasting in skeletal muscles

**DOI:** 10.1002/ctm2.251

**Published:** 2020-12-08

**Authors:** Ji‐Hyung Lee, Hyun‐Jun Kim, Seon‐Wook Kim, JungIn Um, Da‐Woon Jung, Darren R. Williams

**Affiliations:** ^1^ New Drug Targets Laboratory School of Life Sciences Gwangju Institute of Science and Technology Gwangju Republic of Korea

To the Editor:

Skeletal muscle wasting occurs in numerous degenerative diseases associated with high morbidity and mortality and poor capacity for independent living. Thus, it is a major economic burden to society.^1^ Because there are currently no approved drugs for muscle wasting, there is an urgent need to discover new target‐based drugs and therapies.^2^, ^3^ The need for new drugs to treat muscle wasting is increasing due to population aging. Many large pharmaceutical companies have withdrawn from this research due to excessive costs and high‐profile failures, leading to concern about the development of lead compounds for treating muscle wasting.^4^ A potential solution is to repurpose drugs from their original clinical application.^3^ Consequently, our team has identified inositol monophosphatase (IMPase/IMPA1) as a new drug target and the clinically safe drug, ebselen (Figure 1A), as a candidate for drug repurposing. Our data implicate cellular levels of myo‐inositol, a precursor of numerous second messengers,^5^ as a regulator of atrogene expression in muscle wasting.

**FIGURE 1:**
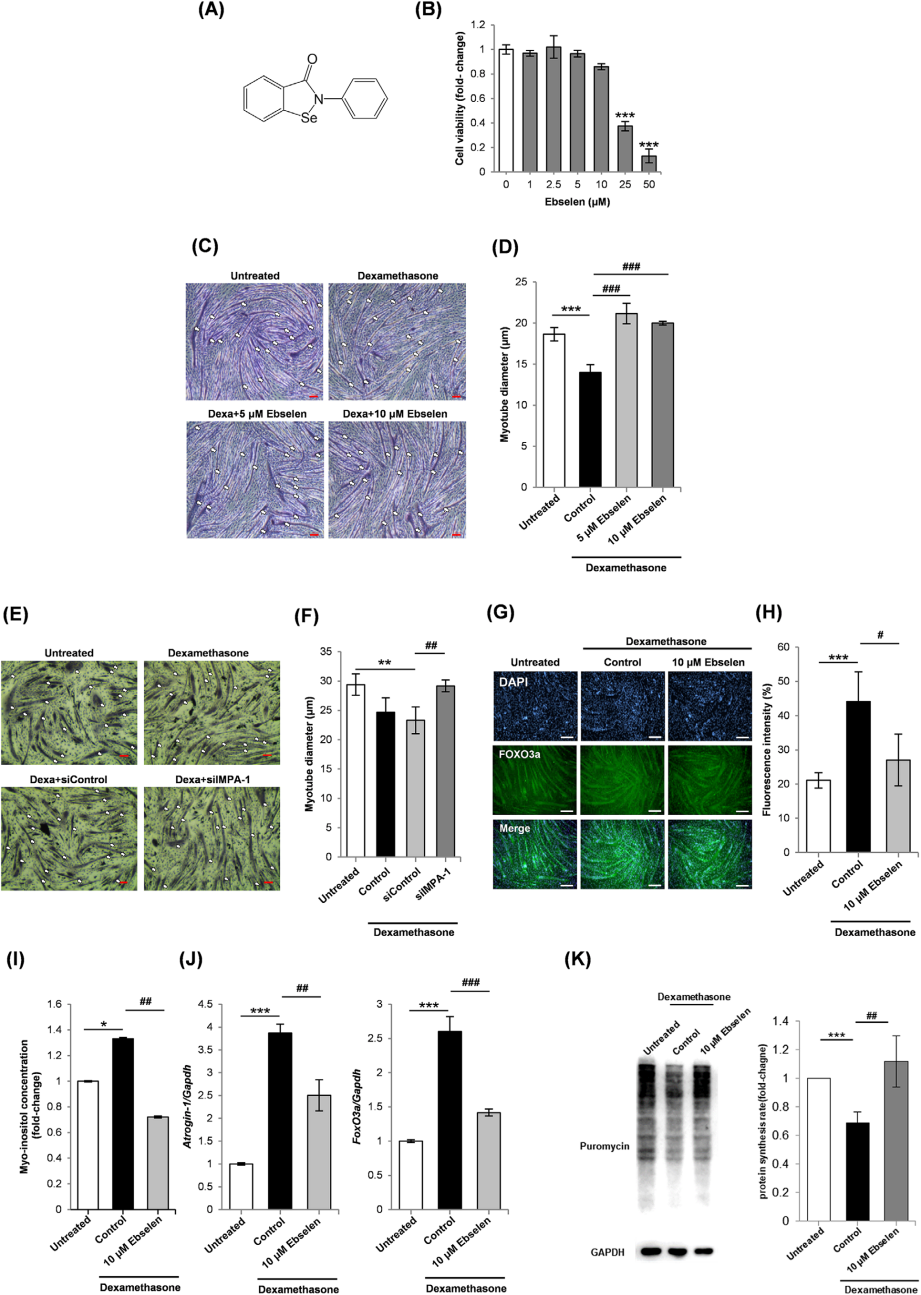
IMPase inhibitor protects against experimentally induced myotube wasting. A, Chemical structure of ebselen, a clinically safe IMPase inhibitor. B, MTT assays for C2C12 myoblasts treated for 48 hours with increasing concentrations of ebselen. IC_50_ = 22.42 μM for ebselen‐treated myoblasts. ***P < .001 when comparing decreased viability in untreated myoblasts. C, Micrographs of H&E‐stained C2C12 myoblasts cultured as follows: (A) differentiation media (DM) for 120 hours (control); (B) DM for 96 hours and DM plus 10 μM dexamethasone (vehicle) for 24 hours; (C) DM for 96 hours and DM plus 10 μM dexamethasone and 5 μM ebselen for 24 hours; (D) DM for 96 hours and DM plus 10 μM dexamethasone and 10 μM ebselen for 24 hours. D, Myotube average diameter. ***P < .001 when comparing the untreated control. ^###^ *P *< .001 when comparing myotubes treated with dexamethasone plus vehicle. E, H&E‐stained C2C12 myoblast cultures after the following treatment conditions: (A) 120‐hour incubation with DM; (B) 96‐hour incubation with DM and 24‐hour treatment with 10 μM dexamethasone; (C) 96‐hour incubation with DM plus control siRNA, and 24‐hour treatment with 10 μM dexamethasone; (D) 96‐hour incubation with DM plus Impa‐1 siRNA, and 24‐hour treatment with 10 μM dexamethasone. The stained myotubes are indicated using white arrows. Scale bar = 100 μm. F, Average myotube diameter. **P < .01 when comparing DM control. ^##^ *P *< .01 when comparing dexamethasone‐treated cultures. G, Immunocytochemistry analysis of FoxO3a expression in C2C12 myoblasts. Myoblasts cultured in DM for 96 hours were treated with 10 μM dexamethasone, or 10 μM dexamethasone and 10 μM ebselen for 24 hours. Scale bar = 100 μm. H, Quantification of FoxO3a staining intensity. ***P < .001 when comparing increased expression in untreated cells. ^#^ *P *< .05 when comparing decreased expression in cells treated with dexamethasone plus vehicle. I, Myo‐inositol level in C2C12 myoblasts cultured as follows: (A) DM for 120 hours; (B) DM for 96 hours and DM plus 10 μM dexamethasone for 24 hours; (C) DM for 96 hours and DM plus 10 μM dexamethasone and 10 μM ebselen for 24 hours. *P < .05 for significantly increased myo‐inositol. ^##^ *P *< .01 for significantly decreased myo‐inositol. J, Representative RT‐PCR analysis of FoxO3a and atrogin‐1 expression in C2C12 myoblasts. Myoblasts cultured in DM for 96 hours were treated with 10 μM dexamethasone, or 10 μM dexamethasone and 10 μM ebselen for 24 hours. ^##^ *P *< .01 and ^###^ *P *< .001 when comparing expression in cells treated with dexamethasone alone. ***P < .001 when comparing expression in cells treated with DM alone. K, SUnSET assay of protein synthesis in C2C12 myoblasts. The myoblasts were cultured in DM for 96 hours and treated with 10 μM dexamethasone, or 10 μM dexamethasone and 10 μM ebselen, for 24 hours. ^##^ *P *< .01 when comparing increased protein expression in cells treated with dexamethasone alone. ***P < .001 when comparing reduced protein synthesis in untreated cells

The effect of targeting IMPase on myogenesis was investigated using three known inhibitors: ebselen, L‐690330, and lithium chloride (LiCl). Cell viability analysis in C2C12 myoblasts and myotubes indicated IC_50_ = 22.42 μM for ebselen‐treated myoblasts and IC_50_ = 19.93 mM for LiCl‐treated myoblasts. L‐690330 treatment did not induce cytotoxicity in C2C12 myoblasts or myotubes (Figure 1B and Figure S1A‐D). Therefore, 10 μM ebselen, 10 μM L‐690330, and 5 mM LiCl were selected. The dexamethasone model was used to measure myotube wasting.^6^ Myotubes treated with ebselen and dexamethasone had higher average diameter and increased proportion of larger myotubes compared to those treated with dexamethasone alone (Figure 1C,D and Figure S1E). To investigate whether IMPase inhibitors produce antiwasting effects, myotubes were treated with dexamethasone alone or in combination with LiCl or L‐690330. Increased overall average myotube diameter and higher proportion of larger diameter myotubes were observed in the cultures treated with LiCl or L‐690330 (Figure S1F‐H). The role of IMPase in wasting was investigated by gene knockdown of IMPase‐1. qPCR and Western blotting showed that IMPase‐1 siRNA treatment reduced expression in C2C12 myoblasts (Figure S2A‐C). IMPase‐1 siRNA prevented myotube wasting caused by dexamethasone and increased the proportion of myotubes with larger diameters (Figure 1E,F and Figure S2D).

Increased skeletal muscle wasting is associated with increased expression of E3 ubiquitin ligases, atrogin‐1 (MAFbx), and MuRF‐1 (TRIM63), which are targets of the master transcription factor, forkhead box O3 (FoxO3a) that is upregulated in muscle wasting.^7^ Immunocytochemistry analysis indicated that FoxO3a expression was increased in dexamethasone‐treated myotubes and decreased in ebselen‐treated myotubes (Figure 1G,H). Myo‐inositol concentration and FoxO3a and atrogin‐1 mRNA expression were increased by dexamethasone and reduced by IMPase inhibitors (Figure 1I,J and Figure S3A‐F). Protein synthesis was measured using SUnSET assay. The IMPase inhibitor ebselen increased protein synthesis in dexamethasone‐treated myotubes (Figure 1K and Figure S3G). The effect of myo‐inositol supplementation on myotube wasting and myogenesis was assessed as previously described.^8^ Myo‐inositol supplementation increased myotube wasting and inhibited myogenesis (Figures S4 and S5A‐D). In contrast, IMPase inhibitors enhanced myogenesis (Figure S5E‐J).

The effect of IMPase inhibition by ebselen in vivo was assessed in the mouse dexamethasone treatment model. Myo‐inositol concentration was increased in the gastrocnemius muscle of mice receiving dexamethasone and lowered in those receiving ebselen (Figure 2A). Dexamethasone and ebselen cotreatment produced a small but significant decrease in body weight compared to untreated mice (Figure S6A). Dexamethasone treatment significantly reduced quadriceps muscle mass, which was recovered by ebselen treatment (Figure 2B). Skeletal muscle performance was assessed using the inverted hanging and grip strength tests. Ebselen monotherapy significantly enhanced hanging time and grip strength compared to dexamethasone monotherapy (Figure 2C,D). Ebselen increased the cross‐sectional area of muscle fiber and the proportion of larger sized fibers (Figure 2E,F and Figure S6B). Expression of FoxO3a target atrogenes, atrogin‐1 and MuRF‐1, in the quadriceps was increased by dexamethasone treatment and reduced by cotreatment with ebselen (Figure S6C). Further assessment of the therapeutic effect of ebselen on muscle wasting was conducted in the glycerol model, which has been used in previous studies of drugs repurposed for muscle wasting.^6^ Ebselen has been approved as an oral medication in humans. Glycerol delivery increased myo‐inositol level in the gastrocnemius muscle and was reduced by ebselen treatment (Figure S7A). Oral ebselen treatment did not significantly affect body weight and had no effect on gastrocnemius mass in the contralateral, nontreated muscle (Figure S7B). Glycerol delivery reduced gastrocnemius and soleus mass, which was increased by ebselen treatment (Figure S7C,D). Ebselen also increased the mass of the noninjected, contralateral soleus muscle (Figure S7E,F). Rotarod testing indicated that ebselen treatment recovered muscle endurance and reduced both muscle fiber damage and cross‐sectional area (Figure S7G‐K). The expression of FoxO3 target atrogenes, atrogin‐1 and MuRF‐1, in the gastrocnemius increased after glycerol injection and was inhibited by ebselen (Figure S7L‐N). To assess whether ebselen has potential as an antiwasting compound in human skeletal muscle, differentiating human primary myoblasts were treated with dexamethasone with or without ebselen. Dexamethasone produced a decrease in both myotube diameter and the proportion of larger diameter myotubes. Ebselen cotreatment inhibited the effects of dexamethasone in the human muscle cells (Figure 3A‐C).

**FIGURE 2:**
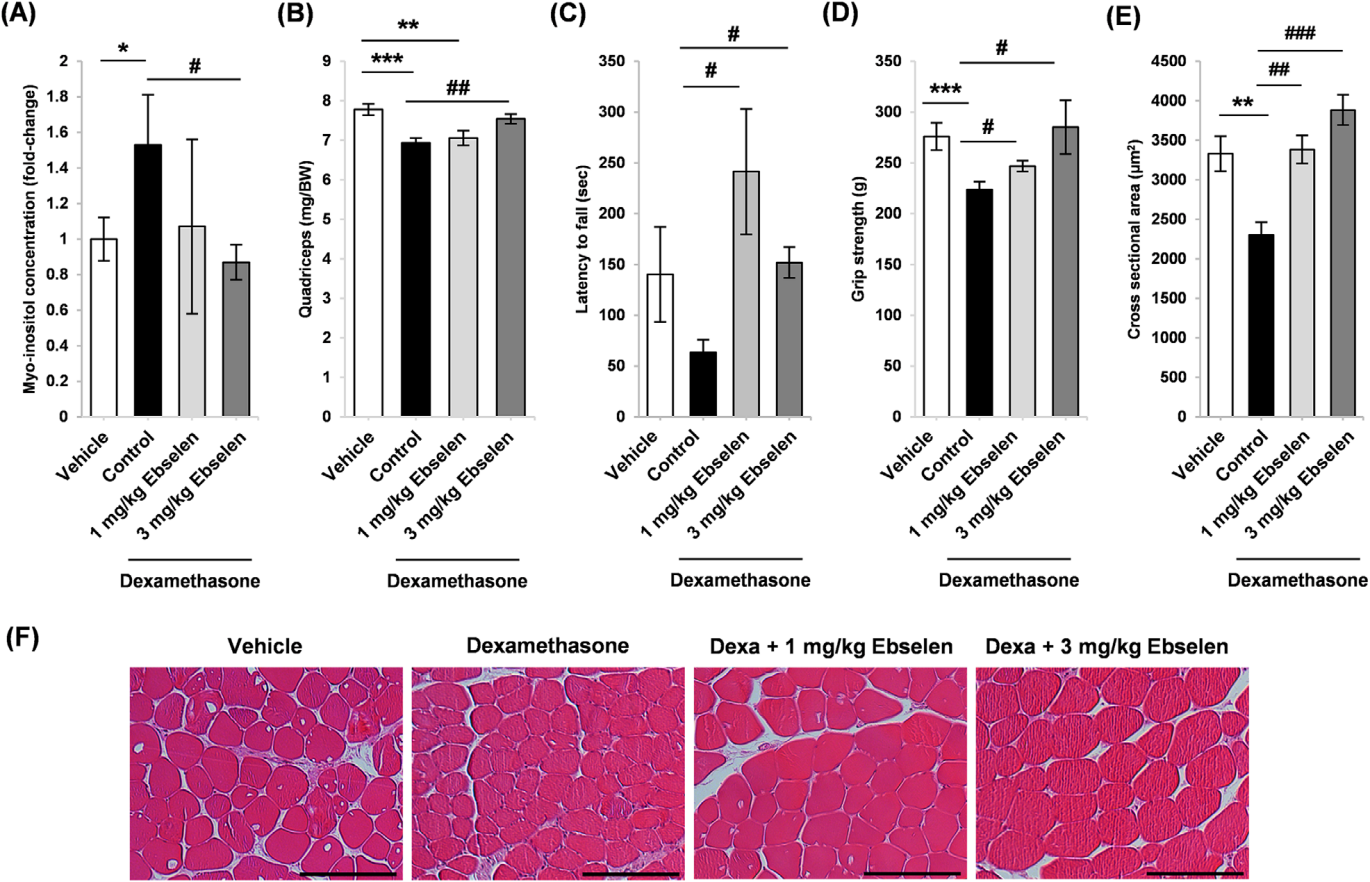
Effect of ebselen in the dexamethasone model of skeletal muscle wasting. A, Myo‐inositol level in the quadriceps muscle of the treated mice. *P < .05 when comparing mice treated with vehicle alone and ^#^ *P *< .05 when comparing mice treated with dexamethasone alone. B, Quadriceps muscle mass (**P < .01 and ***P < .001 when comparing untreated mice; ^##^ *P *< .01 when comparing mice treated with dexamethasone alone). C, Latency to fall off in the hanging tolerance test system (^#^ *P *< .05 when comparing mice treated with dexamethasone alone). D, Grip strength in the treated mice (***P < .001 when comparing untreated mice; ^#^ *P *< .05 when comparing mice treated with dexamethasone plus vehicle). E, Fiber cross‐sectional area distribution in the quadriceps muscle (**P < .01 when comparing untreated mice; ^##^ *P *< .01 and ^###^ *P *< .001 when comparing mice treated with dexamethasone alone). F, Representative images of H&E‐stained quadriceps muscle. Scale bar = 200 μm

**FIGURE 3:**
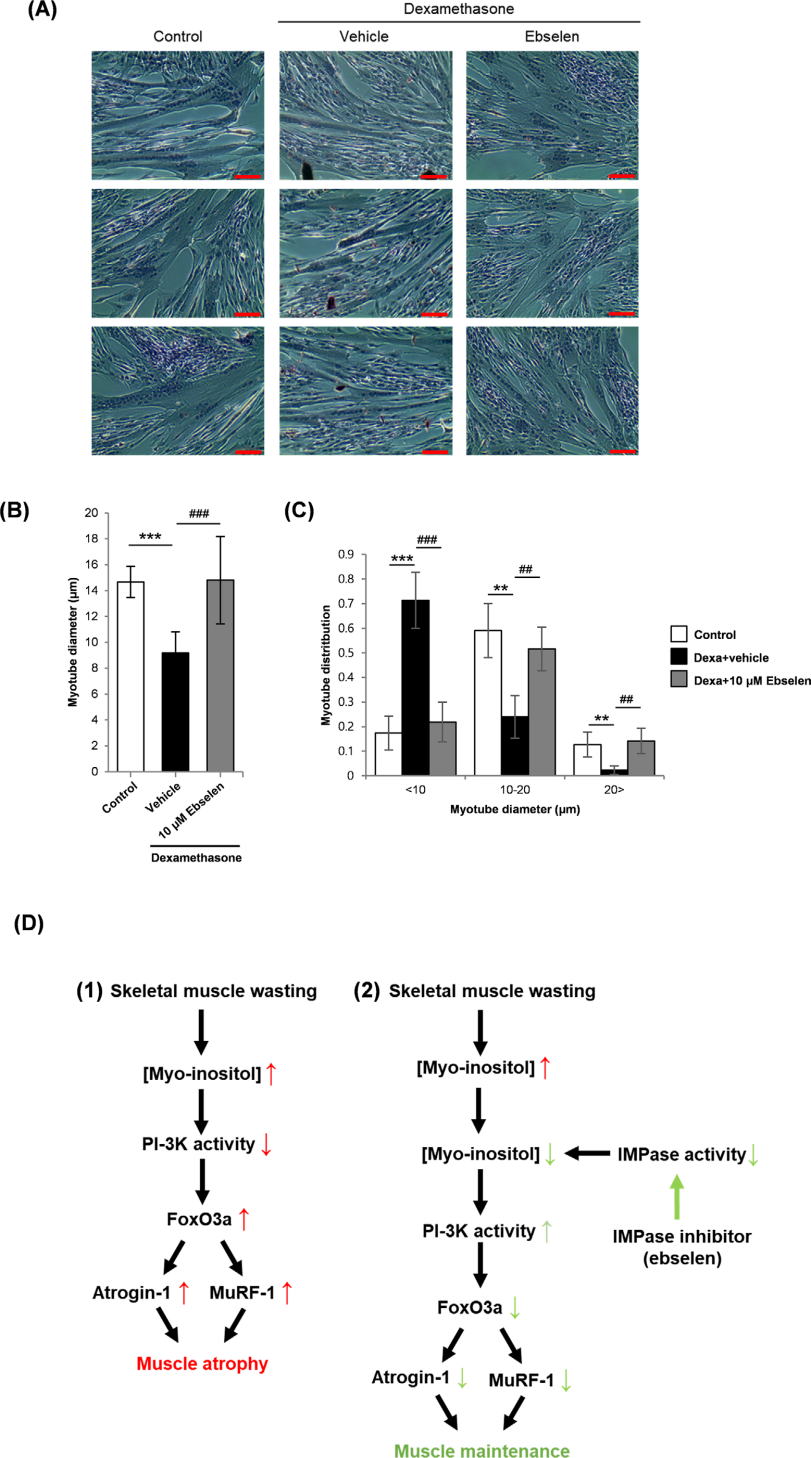
Effect of ebselen on muscle wasting in human skeletal myotubes. A, DIC images of human skeletal myoblasts cultured as follows: (A) DM for 96 hours (control); (B) DM for 72 hours and DM plus 10 μM dexamethasone for 24 hours; (C) DM for 72 hours and DM plus 10 μM dexamethasone and 10 μM ebselen for 24 hours. Scale bar = 100 μm. B and C, Myotube average diameter and myotube diameter distribution. **P < .01 and ***P < .001 for comparing the control. ^##^ *P *< .01 and ^###^ *P *< .001 for comparing myoblasts treated with dexamethasone plus vehicle. D, Model for the mechanism of action of IMPase inhibition to protect from muscle wasting. In scheme (1), a muscle wasting stimulus such as dexamethasone or glycerol treatment, as used in this study, produces increased intracellular concentrations of myo‐inositol. Excess myo‐inositol levels have previously been shown to inhibit PI3K signaling, which increases the expression of FoxO3a and the target atrogenes, atrogin‐1 and MuRF‐1, initiating skeletal muscle wasting.^9^, ^10^ In scheme (2), treatment with an IMPase inhibitor at the onset of muscle wasting blocks the increase in myo‐inositol, leading to suppression of atrogene induction and preservation of muscle mass

In conclusion, here in this study, we provide evidence that IMPase inhibition, modulation of myo‐inositol concentration, and repurposing of the clinically safe drug ebselen may be a novel drug development strategy for skeletal muscle wasting (Figure 3D). Ebselen was studied in numerous clinical trials as a treatment for psychological disorders, while our results presented ebselen as a new alternative and described its potential for improving muscle wasting. The identification of IMPase as a potential new target for drug discovery can also facilitate efforts to develop novel chemical entities for the effective treatment of muscle wasting disorders.

## AUTHOR CONTRIBUTIONS

Ji‐Hyung Lee and Hyun‐Jun Kim carried out cell‐based and animal experiments, and analyzed the data. Seon‐Wook Kim, and JungIn Um assisted Ji‐Hyung Lee with animal experiments. Da‐Woon Jung and Darren R. Williams designed the study and wrote the manuscript.

## CONFLICT OF INTEREST

Darren R. Williams, Da‐Woon Jung, Ji‐Hyung Lee, and Hyun‐Jun Kim have applied for a patent concerning the use of organoselenium compounds to treat skeletal muscle wasting.

## Supporting information

Supporting Information

## References

[1] Ding S , Dai Q , Huang H , Xu Y , Zhong C . An overview of muscle atrophy. Adv Exp Med Biol. 2018;1088:3‐19. 30390245 10.1007/978-981-13-1435-3_1

[2] Um J , Jung DW , Williams DR . Lessons from the swamp: developing small molecules that confer salamander muscle cellularization in mammals. Clin Transl Med. 2017;6:13. 28332147 10.1186/s40169-017-0143-8 PMC5362566

[3] Kim WH , Shen H , Jung DW , Williams DR . Some leopards can change their spots: potential repositioning of stem cell reprogramming compounds as anti‐cancer agents. Cell Biol Toxicol. 2016;32:157‐168. 27156576 10.1007/s10565-016-9333-1

[4] Garber K . No longer going to waste. Nat Biotechnol. 2016;34:458‐461. 27153267 10.1038/nbt.3557

[5] Parthasarathy LK , Seelan RS , Tobias C , Casanova MF , Parthasarathy RN . Mammalian inositol 3‐phosphate synthase: its role in the biosynthesis of brain inositol and its clinical use as a psychoactive agent. Subcell Biochem. 2006;39:293‐314. 17121280 10.1007/0-387-27600-9_12

[6] Chiu HC , Chiu CY , Yang RS , Chan DC , Liu SH , Chiang CK . Preventing muscle wasting by osteoporosis drug alendronate in vitro and in myopathy models via sirtuin‐3 down‐regulation. J Cachexia Sarcopenia Muscle. 2018;9:585‐602. 29512306 10.1002/jcsm.12289 PMC5989760

[7] Sandri M , Sandri C , Gilbert A , et al. Foxo transcription factors induce the atrophy‐related ubiquitin ligase atrogin‐1 and cause skeletal muscle atrophy. Cell. 2004;117:399‐412. 15109499 10.1016/s0092-8674(04)00400-3 PMC3619734

[8] Slaby F , Bryan J . High uptake of myo‐inositol by rat pancreatic tissue in vitro stimulates secretion. J Biol Chem. 1976;251:5078‐5086. 956177

[9] Koguchi T , Tanikawa C , Mori J , Kojima Y , Matsuda K . Regulation of myo‐inositol biosynthesis by p53‐ISYNA1 pathway. Int J Oncol. 2016;48:2415‐2424. 27035231 10.3892/ijo.2016.3456

[10] Unver N , Delgado O , Zeleke K , et al. Reduced IL‐6 levels and tumor‐associated phospho‐STAT3 are associated with reduced tumor development in a mouse model of lung cancer chemoprevention with myo‐inositol. Int J Cancer. 2018;142:1405‐1417. 29134640 10.1002/ijc.31152 PMC5805587

