# Supplementary material for: Inhibited inositol monophosphatase and decreased myo‐inositol concentration improve wasting in skeletal muscles
Source: Clin Transl Med. 2020 Dec 8;10(8):e251. doi: 10.1002/ctm2.251 (PMC7724229; doi:10.1002/ctm2.251)
Supplement: Supplementary file 1 — Supporting Information [file CTM2-10-e251-s001.docx]

**Targeting inositol monophosphatase and elevated myo-inositol inhibits wasting in skeletal muscle**

Ji-Hyung Lee, Hyun-Jun Kim, Seon-Wook Kim, JungIn Um, Da-Woon Jung and Darren R. Williams

New Drug Targets Laboratory, School of Life Sciences, Gwangju Institute of Science and Technology, 1 Oryong-Dong, Buk-Gu, Gwangju 61005, Republic of Korea

**Supporting Information**

**Methods**

**Reagents**

Dexamethasone and L-690, 330 were purchased from Santa Cruz Biotechnology, USA. Ebselen was purchased from Tokyo Chemical Industry Co. Ltd., Japan. MitoTracker Red CMX-Ros was purchased from Invitrogen, USA. Lithium chloride (LiCl) was purchased from Sigma-Aldrich, USA. 6-Bromoindirubin-3-oxime (BIO) was a kind gift from Professor Yong-Chul Kim, Gwangju Institute of Science and Technology, Republic of Korea. Myo-inositol was purchased from MP Biomedicals, USA. Puromycin was purchased from Abcam, UK. Glycerol was purchased from Wako Chemicals, Japan. Antibodies were purchased for myosin heavy chain 2 (sc-53095, Santa Cruz Biotechnology, USA; dilution=1:1000), forkhead box O3 (FoxO3a) (12829S, Cell Signaling Technology, USA; immunoblotting dilution=1:1000, immunocytochemistry dilution=1:400), glyceraldehyde 3-phosphate dehydrogenase (GAPDH) (sc-365062, Santa Cruz Biotechnology; dilution=1:1000), IMPase1 (ab202131,Abcam; dilution=1:1000) and puromycin (MABE343, Millipore, USA; dilution=1:25000).

**Cell culture**

C2C12 murine skeletal muscle precursor cells (myoblasts) were maintained in growth media (GM), consisting of Dulbecco’s Modified Eagle’s Medium (DMEM) supplemented with 10% fetal bovine serum (FBS), 50 units/mL penicillin and 50 mg/mL streptomycin (PenStrep). Myoblasts were induced to differentiate into myotube by treatment with differentiation media (DM: DMEM supplemented with 2% horse serum (HS) and PenStrep) for 96 h.

**Myoblast differentiation and myotube treatment**

C2C12 myoblasts were seeded onto 6-well plates in GM. After reaching confluence, the cultures were treated with DM for 96 h, followed by DM containing 10 μM dexamethasone to induce myotube wasting, with or without drug of interest, for 24 h. Myotubes were visualized using hematoxylin and eosin staining, and imaged with light microscopy (Olympus CKX41, Japan). Myotube distribution was calculated as the number of myotubes in each group divided by the total number of myotubes per picture.

**MTT assay**

Cell viability was assessed using the MTT (3-(4,5-dimethylthiazol-2-yl)-2,5-diphenyltetrazolium bromide) assay, as previously described [[1](#_ENREF_1), [2](#_ENREF_2)]. Myoblasts were seeded in triplicate in a 96-well plate at a density of 2 x 10^3^ cells per well. Compound of interest was added and, after 48 h incubation, the medium was changed to MTT solution (0.5 mg/mL, final concentration) and the plate was incubated in a 37°C 5% CO_2_ incubator. After 60 min incubation, 50 μL of DMSO was added. Optical density in each well was measured at 570 nm with a microplate reader (VersaMax, Molecular Devices, USA). For myotube analysis, the myoblasts were induced to undergo differentiation for 96 h, after which the media was replenished with compound of interest, and the cultures were incubated for a further 24 h before commencing the assay.

**Myo-inositol assay**

Myo-inositol level in cells and tissues was measured using the Myo-inositol Assay Kit (Megazyme, Ireland), following the manufacturer’s instructions. Skeletal muscle tissue samples and cell supernatants were prepared by sonication in PBS for 1 min 30 sec with a 30 sec on/15 sec off cycle (Vibra-Cell, USA) followed by centrifugation at 13,000 × *g* for 15 min, as previously described [[3](#_ENREF_3)].

**siRNA mediated gene knockdown**

siRNA-mediated knockdown of gene expression was carried out in accordance with the manufacturer’s protocol in the 6-well plate format (Thermo Fischer Scientific, Waltham, USA). The Lipofectamine 3000 agent was used for the transfection step (Thermo Fischer Scientific, Waltham, USA).

**Real-time quantitative PCR**

The transcript level of genes of interest was measured using the StepOnePlus Real Time PCR System (Applied Biosystems, UK). cDNA was reverse transcribed from the total RNA using the AccuPower® RT PreMix (Bioneer, USA). Real-time PCR (qPCR) was carried out according to the manufacturer’s instructions with the following modifications: PCR was performed in triplicate in a total volume of 20 μL 2X Power SYBR® Green PCR Master Mix (Enzynomics, Korea) containing 200 nM (final concentration) of the specific primer and 1 μL of cDNA. PCR amplification was preceded by incubation for 10 min at 95 °C and the amplification step consisted of 40 cycles of denaturation (15 s at 95 °C), annealing (1 m at 60 °C) and extension (72 °C for 20 s) The extension was performed with fluorescence detection at 72 °C after each cycle. After the final cycle, melting-point analysis the samples was performed within the range of 60–95 °C with continuous fluorescence detection. A specific cDNA sample was included in each run and served as a reference for the comparison between runs. The expression level of GAPDH was used for normalization while calculating the expression levels of all of the other genes (as indicated in the text). Details of the primers are showed in Table 1.

**Table 1:** Primers used in this study.

| **Gene** | **Accession number** | **Direction** | **Sequence** |
| --- | --- | --- | --- |
| Mus musculus myogenic factor 5 (Myf5) | NM_008656 | Forward | AGCTGGGCAGAATACGTGCTT |
|  |  | Reverse | AGAACAGGCAGAGGAGAATCCA |
| Mus musculus paired box 7 (Pax7) | NM_011039 | Forward | CCCTTTCAAAGACCAAATGCA |
|  |  | Reverse | CCCTCACGGGCAGATCATTA |
| Mus musculus myogenin (Myog) | NM_031189 | Forward | AGCGCAGGCTCAAGAAAGTG |
|  |  | Reverse | CCGCCTCTGTAGCGGAGAT |
| Mus musculus glyceraldehyde-3-phosphate dehydrogenase (Gapdh) | NM_001289726 | Forward | CTCCACTCACGGCAAATTCA |
|  |  | Reverse | GCCTCACCCCATTTGATGTT |
| Mus musculus myosin, heavy polypeptide 2, skeletal muscle, adult (Myh2) | NM_001039545 | Forward | GATCACCACGAACCCATATGATT |
|  |  | Reverse | TTCATGTTCCCATAATGCATCAC |
| Mus musculus forkhead box O3 (Foxo3) | NM_019740 | Forward | TGGAGTCCATCATCCGTAGTGA |
|  |  | Reverse | CTGGTACCCAGCTTTGAGATGAG |
| Mus musculus inositol (myo)-1(or 4)-monophosphatase 1 (Impa1) | NM_018864 | Forward | AGCTGTTTCAATTGGCTTCCTT |
|  |  | Reverse | GCCGGTGTACATCTTATCTTCCA |
| Mus musculus inositol (myo)-1(or 4)-monophosphatase 2 (Impa2) | NM_053261 | Forward | TCCCCACTGTGGCAGTTAGC |
|  |  | Reverse | CCCTCCTGCCGGTGTACA |

**MitoTracker Red CMX-Ros staining of early stage myotubes**

Myotubes in differentiating cultures were visualized by treatment with 50 nM MitoTracker Red CMX-Ros and 1 μM DAPI for 30 min at 37 °C in differentiation media, as previously described [[4](#_ENREF_4)], and imaged using fluorescent microscopy (Leica DMI3000B, Germany).

**Morphological analysis of myotubes**

To assess myotube formation and wasting, five microscopic fields of H&E or MitoTracker Red CMX-Ros stained cultures were captured randomly to calculate multinucleated myotube formation and diameters, as previously described [[5](#_ENREF_5)]. Myotubes were designated as multinucleate cells containing three or more nuclei and myotube diameters were calculated by using the ImageJ 1.48 software (National Institutes of Health, Bethesda, MD, USA).

**Western blotting**

Cell lysate protein concentration was quantified using the Bradford reagent (Bio-Rad, USA, CA). After electrophoresis, separated proteins were transferred on to PVDF membranes, blocked with 5% non-fat powdered milk in TBST (0.02% Tween 20 in TBS) and 5% bovine serum albumin in TBST (0.02% Tween 20 in TBS) and subsequently incubated overnight at 4°C with the primary antibody of interest. The secondary antibody was used at a 1: 10000 dilution, with incubation for 35 min at room temperature. Densitometry analysis of the gel bands was carried out using ImageJ 1.48 software (National Institutes of Health).

**Immunocytochemistry**

Myoblasts were differentiated into myotubes in 6 well plates. Myotubes were then immunostained for FoxO3a (Cell Signaling Technology; dilution1:400). Alexa Fluor 488 goat anti-mouse IgG was used as a secondary antibody (Invitrogen). Nuclei were stained using DAPI solution (1 μM dissolved in 3rd distilled water). Staining was visualized by fluorescent microscopy (Leica DMI3000 B).

**Surface sensing of translation (SUnSET) assay of protein synthesis *in vitro***

The SUnSET assay was carried out as previously described [[6](#_ENREF_6)]. In brief, 1 μg/mL puromycin was added to the cultures and lysates harvested 10 min later. Myotube extracts were processed for immunoblotting using an anti-puromycin 12D10 antibody (MABE343; Millipore).

**Animal studies**

Studies were carried out in accordance with the Institute for Laboratory Animal Research Guide for the Care and Use of Laboratory Animals and were approved by the Gwangju Institute of Science and Technology Animal Care and Use Committee (study approval number GIST-2019-042). Mice were supplied by Damool Science, Republic of Korea.

**Dexamethasone model of skeletal muscle wasting**

Treatment with the glucocorticoid dexamethasone is a commonly employed model of skeletal muscle wasting [[7](#_ENREF_7), [8](#_ENREF_8)]. 13 week old male C57BL/6J mice were treated with drugs as follows: 1) Injection of vehicle (4% hydroxypropyl-β-cyclodextrin) alone, 2) 15 mg/kg dexamethasone dissolved in vehicle, 3) Injection of 15 mg/kg dexamethasone and 1 mg/kg ebselen, 4) Injection of 15 mg/kg dexamethasone and 3 mg/kg ebselen (n=5 per group). The dose of ebselen was based on the study by Antoniadou, *et al* [[9](#_ENREF_9)]. Mice were treated daily by intraperitoneal injection for 14 days and then assessed for muscle condition.

**Glycerol model of skeletal muscle degeneration**

Intramuscular glycerol injection in mice produces adipogenesis in addition to fibrous scar formation, which can be observed in some types of muscle wasting [[10](#_ENREF_10)]. Male C57BL/6J mice received an intramuscular injection of 100 μL glycerol (50% vol/vol) into monolateral gastronemius and soleus muscle under anaesthesia induced by ketamine (22 mg/kg; Yuhan, Republic of Korea) and xylazine (10 mg/kg; Bayer, Republic of Korea) in PBS. The volume of glycerol was based on a previous study by Chiu, *et al* [[5](#_ENREF_5)]. Mice were treated with vehicle (0.5% methylcellulose) or vehicle plus ebselen (30 mg/kg) via oral gavage every 24 h (n=6 per group). The dose of ebselen was based on the previous study by Haddad el, *et al* [[11](#_ENREF_11)]. The muscle fatigue test was carried out after 5 days.

**Muscle fatigue test**

Muscle fatigue was measured using the protocol previously described by Chiu, et al [[5](#_ENREF_5)]. In brief, the mice were accommodated to training before the commencement of the fatigue task using an accelerating rotarod (Ugo Basile, Italy). The mice were trained with the rotarod at a constant speed of 13 rpm for 15 min. After 15 min recovery, the mice were placed on the rotarod adjusted to accelerate from 13 to 25 rpm in 3 min for 15 min. 24 h later, the muscle fatigue test was carried out speeds ramping from 13 to 25 rpm in 3 min and maintained at 25 rpm for a 30 min interval. Latency to fall off the rotarod for each mouse was measured. A fatigued mouse was classified as falling off 4 times within 1 min, which terminated the test.

**Grip strength test**

Mice grip strength was recorded by using BIO-GS3 (Bioseb, USA). Mice were placed on the metal grid with four paw and pulled softly backward to measure the grip strength until mice cannot keep holding the grid. Maximum value obtained from 3 trials with a 1 min interval was used to represent muscle force.

**Four limb hanging test of muscle strength and endurance**

Mice were placed on the middle of a grid and checked they grasped the grid with four limbs. The grid was then inverted with a 50 cm height above the baseline, and the hanging time was measured up to 10 min.

**Muscle sampling and histological analysis**

Mice were anesthetized using ketamine (22 mg/kg; Yuhan, Republic of Korea) and xylazine (10 mg/kg; Bayer, Republic of Korea) in PBS by intraperitoneal injection before sacrifice. In the glycerol injury model, gastrocnemius and soleus muscles were dissected and weighed. For histological analysis, gastrocnemius muscles were fixed by overnight incubation with 4% paraformaldehyde at 4 ℃ and embedded into paraffin. 5 µm muscle sections were obtained and used for H&E staining with a kit, following the manufacturer’s instructions (Merck & Co., USA). Damaged area and cross sectional area was measured with ImageJ 1.48 software (National Institutes of Health). For the dexamethasone induced wasting model, the quadriceps, gastrocnemius and soleus muscles were dissected and weighed. Quadriceps muscles were used for histological analysis with H&E staining, following the same protocol used in glycerol experiment.

**Human skeletal myoblasts culture and experimentation**

Human skeletal myoblasts were purchased from Thermo-Fisher Scientific. Myoblasts were thawed in a water bath, washed with 20 mL DM and centrifuged at 180 *g* for 5 min at room temperature. The myoblasts were re-suspended in DM and seeded onto 12 wells plates and a density of 4.8 x 10^4^ cells/well. 72 h later, myoblasts were treated with compounds for 24 h. Myotube diameter was measured by light microscopy analysis of DIC captured images (Olympus CKX41).

**Statistics**

The Student’s *t*-test was used to determine statistical significance in Figures 1-6 and Figure 9 (Microsoft Excel 2016). Two-way ANOVA was used to determine statistical significance in Figures 7 and 8 (ANOVA was carried out using the Data Analysis ToolPak for Microsoft Excel 2013). *p* values of less than 0.05 were deemed to be statistically significant. Unless otherwise stated, experiments were carried out in triplicate and the error bars are standard deviation.

**Supporting Figures**

**
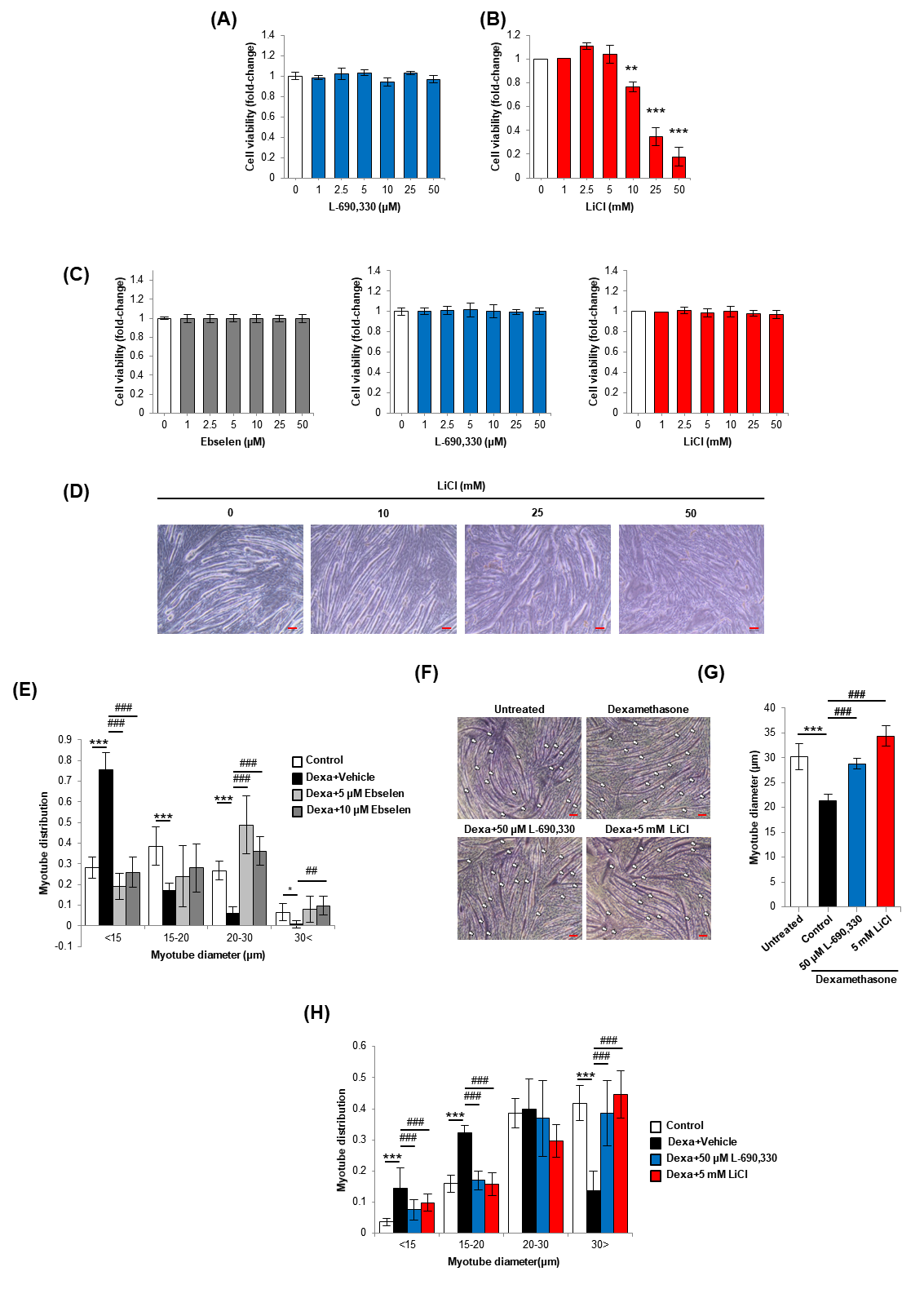
**

**Supporting Figure 1.** A-B, MTT assays for C2C12 myoblasts treated for 48 h with increasing concentrations of LiCl or L-690, 330. IC_50_=19.93 mM for LiCl treated myoblasts. **=*p*<0.01 and ***=*p*<0.001 for decreased viability compared to untreated myoblasts. C, MTT assays for C2C12 myotubes treated with increasing concentrations of LiCl or L-690, 330. D, Phase-contrast micrographs of C2C12 myotubes treated with 10, 25 or 50 mM LiCl for 72 h. Abnormal myotubes can be observed at the higher treatment concentrations. Scale bar=100 µm. E, Myotube diameter distribution in C2C12 myoblasts cultured as follows: 1) DM for 120 h (control), 2) DM for 96 h and DM plus 10 µM dexamethasone (Vehicle) for 24 h, 3) DM for 96 h and DM plus 10 µM dexamethasone and 5 µM ebselen for 24 h, 4) DM for 96 h and DM plus 10 µM dexamethasone and 10 µM ebselen for 24 h. *=*p*<0.05 and ***=*p*<0.001 for significantly different compared to the untreated control. ^##^=*p*<0.01 and ^###^=*p*<0.001 for significantly different compared to treatment with dexamethasone and vehicle. F, Micrographs of H&E stained C2C12 myoblast cultures after 96 h incubation with DM and 24 h treatment with 10 µM dexamethasone, 10 µM dexamethasone and 50 µM L-690, 330, or 10 µM dexamethasone and 5 mM LiCl. The stained myotubes are indicated using white arrows. Scale bar=100 µm. G, Myotube average diameter. H, Myotube diameter distribution. ***=*p*<0.001 for significantly different compared to the untreated control. ^###^=*p*<0.001 for significantly different compared to treatment with dexamethasone plus vehicle.

**
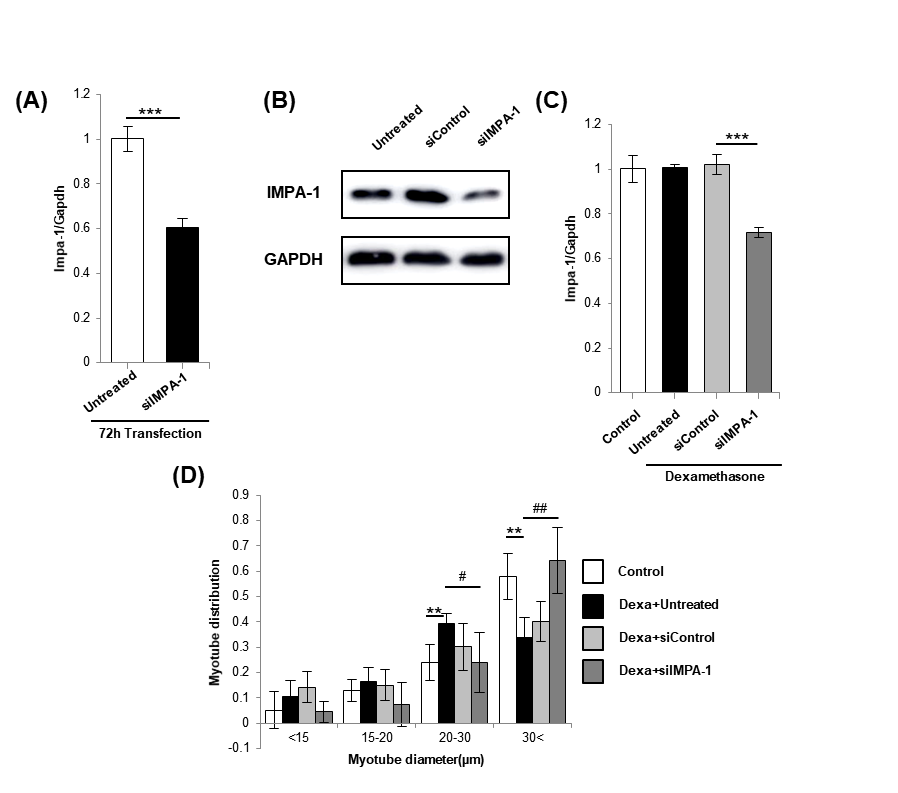
**

**Supporting Figure 2.** A-C, qPCR and western blot confirmation of Impa-1 gene knockdown by siRNA. ***=*p*<0.001 for significantly decreased expression. D, Myotube diameter distribution in C2C12 myoblast cultures after the following treatment conditions: 1) 120 h incubation with DM, 2) 96 h incubation with DM and 24 h treatment with 10µM dexamethasone, 3) 96 h incubation with DM plus control siRNA, and 24 h treatment with 10 µM dexamethasone, 4) 96 h incubation with DM plus Impa-1 siRNA, and 24 h treatment with 10 µM dexamethasone. **=*p*<0.01 for significant difference compared to DM control. #=*p*<0.05 and ##=*p*<0.01 for significant difference compared to dexamethasone treated cultures.


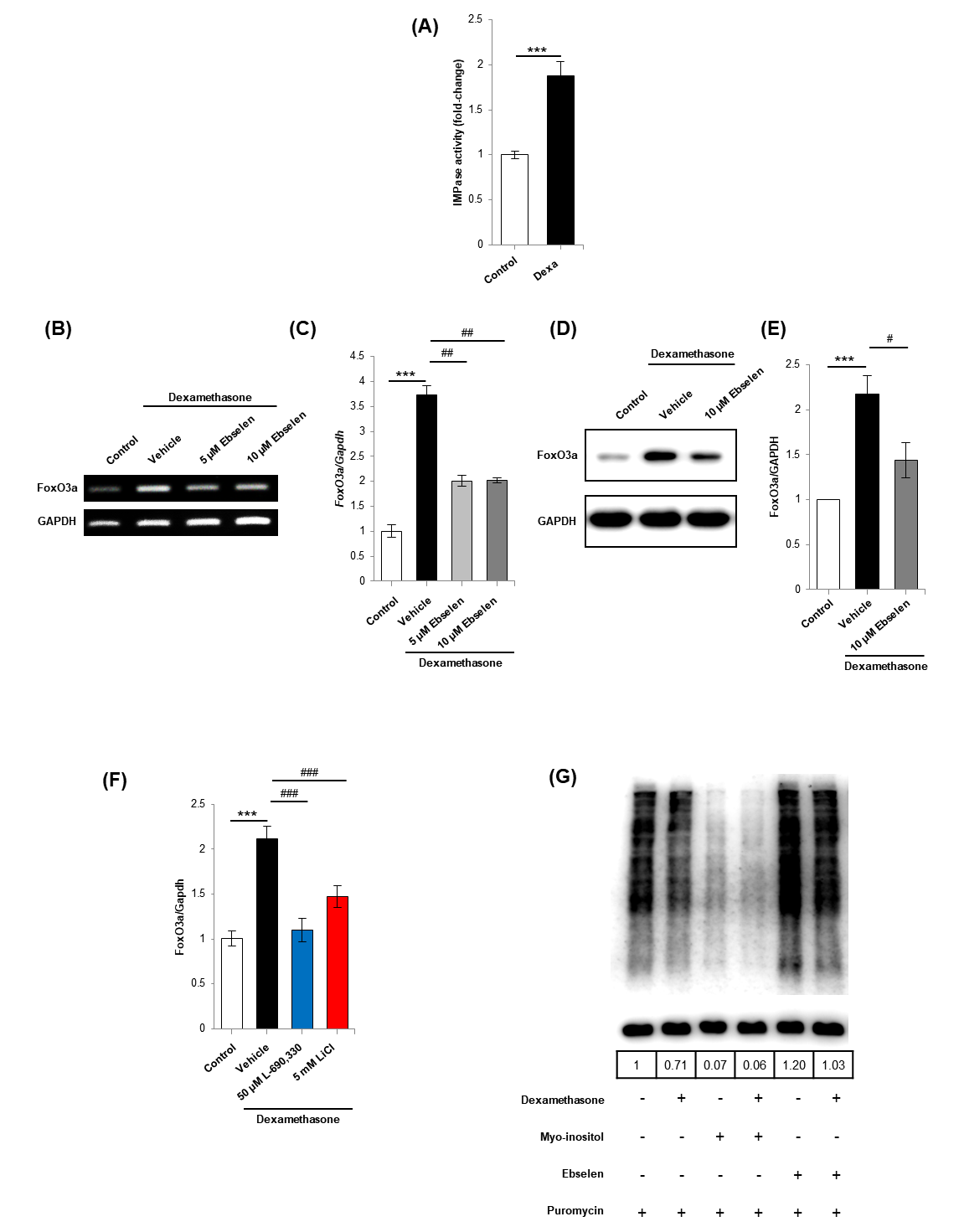


**Supporting Figure 3.** A, Myo-inositol level in C2C12 myoblasts cultured in DM for 72 h (control) or cultured in DM for 72 h and treated with 10 µM dexamethasone for 24 h. ***=*p*<0.001 for significantly increased myo-inositol. B, Representative RT-PCR analysis of FoxO3a expression in C2C12 myoblasts. Myoblasts cultured in DM for 96 h were treated with 10 µM dexamethasone, or 10 µM dexamethasone and 10 µM ebselen, for 24 h. C, qPCR analysis of FoxO3a expression in the treated C2C12 myoblasts. ^##^=*p*<0.01 for significantly different expression compared to dexamethasone alone. ^***^=*p*<0.001 for significantly different expression compared to DM alone. D-E, Western bot analysis of FoxO3a expression. ^#^=*p*<0.05 for significantly different expression compared to dexamethasone alone. ^***^=*p*<0.001 for significantly different expression compared to DM alone. F, qPCR analysis of FoxO3a expression in C2C12 myoblasts treated with dexamethasone and IMPase inhibitors. Myoblasts cultured in DM for 96 h were treated with 10 µM dexamethasone, or 10 µM dexamethasone and 50 µM L-690, 330, or 10 µM dexamethasone and 5 mM LiCl, for 24 h. ***=*p*<0.01 for significantly increased expression compared to the DM alone control. ^###^=*p*<0.001 for decreased expression compared to dexamethasone treatment. G, SUnSET assay of protein synthesis in C2C12 myoblasts cultured in DM for 96 h and treated with 10 µM dexamethasone alone, myo-inositol alone, 10 µM dexamethasone plus myo-inositol, 10 µM ebselen, 10 µM dexamethasone plus 10 µM ebselen, or 10 µM dexamethasone plus 5 mM LiCl, for 24 h. Numbers beneath each lane show the band intensity for puromycin labeled proteins.

**
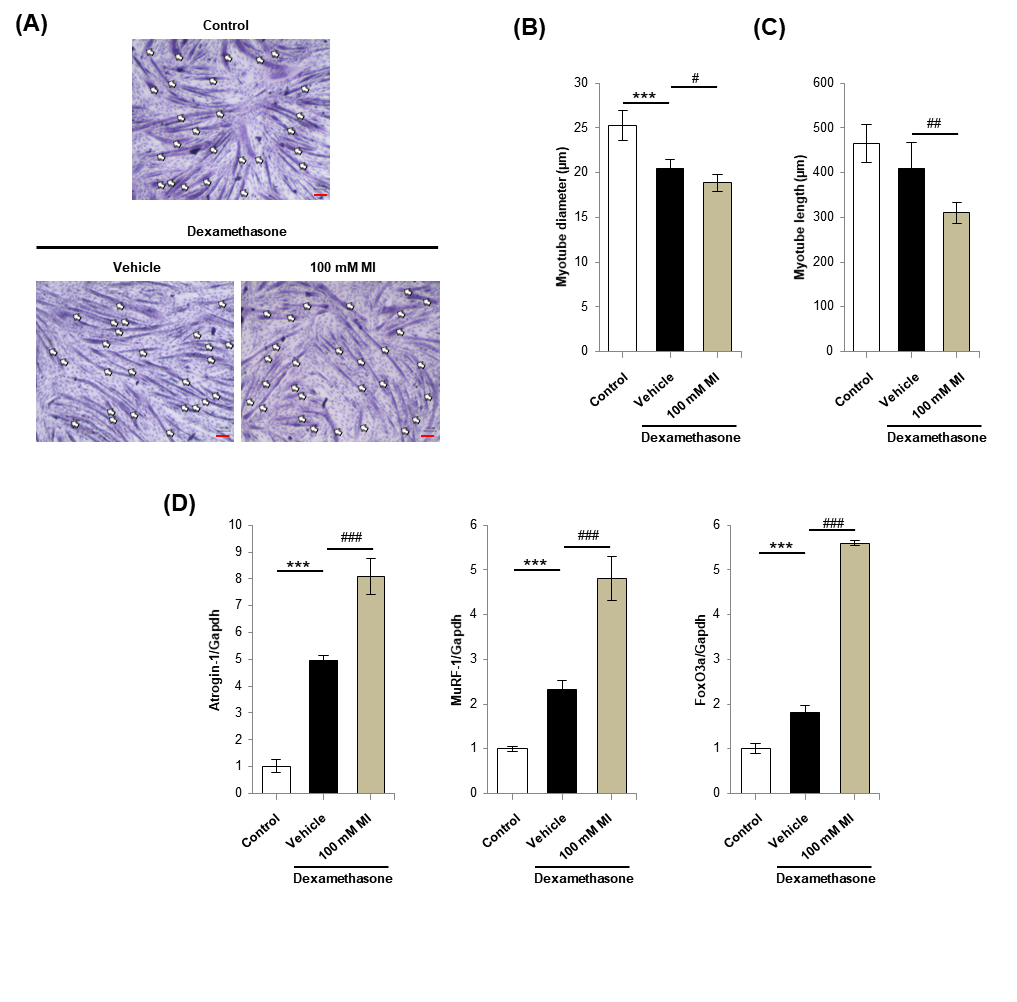
**

**Supporting Figure 4.** A, Micrographs of H&E stained C2C12 myoblast cultures after 96 h incubation with DM and 24 h treatment with 10 µM dexamethasone, or 10 µM dexamethasone plus 100 mM myo-inositol. The stained myotubes are indicated using white arrows. Scale bar=100 µm. B, Average myotube diameter in the treated cultures. ***=*p*<0.001 for decreased average diameter compared to untreated cultures. #=*p*<0.05 for decreased average diameter compared to dexamethasone treated cultures. C, Average myotube length in the treated cultures. ##=*p*<0.01 for decreased average length compared to untreated cultures. D, qPCR analysis of the expression of atrogin-1, MuRF-1 and FoxO3a. ***=*p*<0.001 for increased expression compared to control cultures. ^###^=*p*<0.001 for increased expression compared to dexamethasone treated cultures.


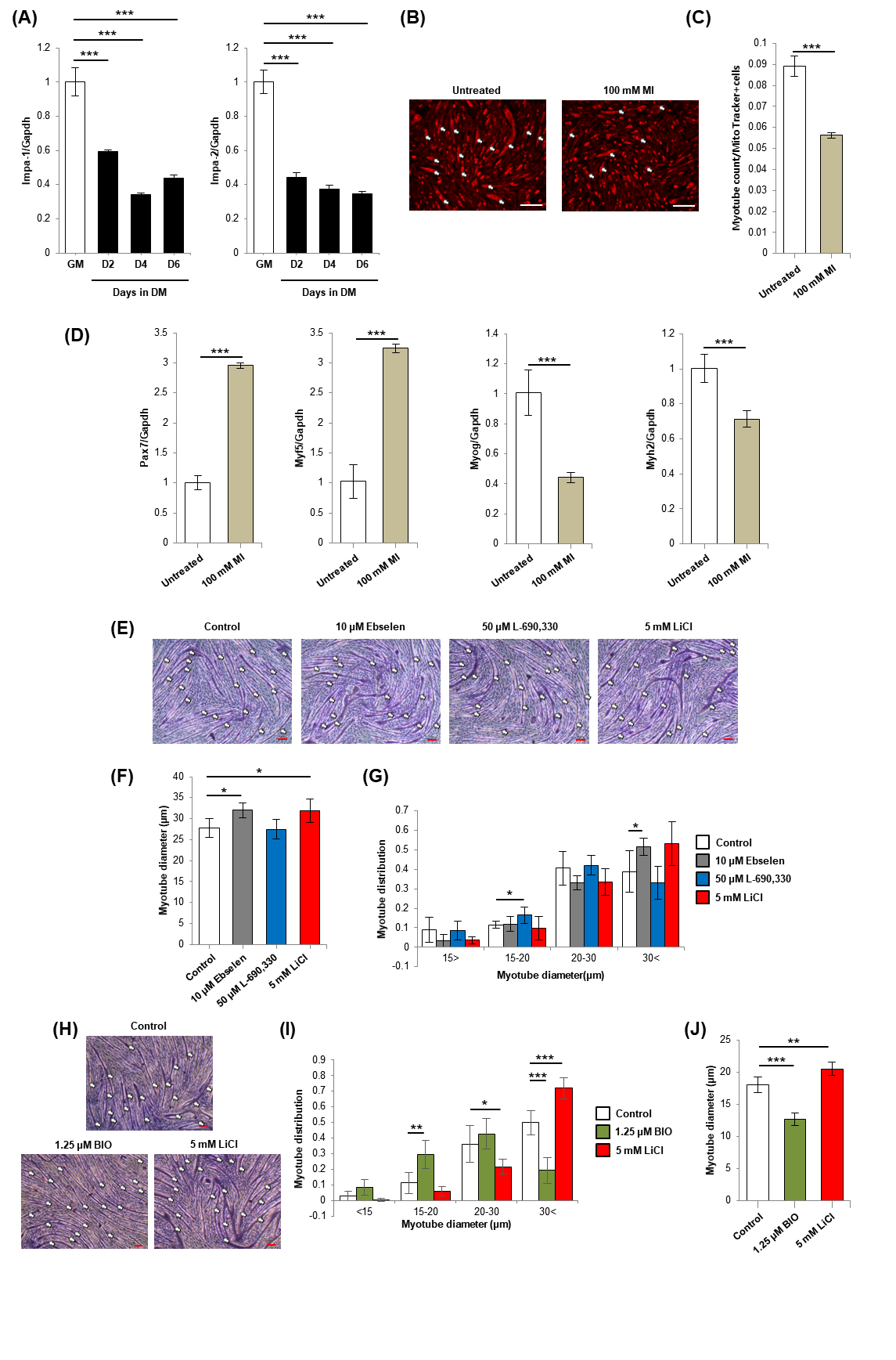


**Supporting Figure 5.** A, qPCR analysis of IMPase1 and IMPase2 expression in C2C12 myoblasts treated with DM for 6 d. ***=*p*<0.01 for decreased expression compared to GM treatment. B, MitoTracker Red CMX-Ros stained C2C12 myoblast cultures after 48 h incubation with DM, or DM containing 100 mM myo-inositol (MI). Scale bar=100 µm. C, Relative fusion index in the cultures. ***=*p*<0.001 for decreased number of myotubes compared to untreated cultures. D, qPCR analysis of Pax7, Myf5, MyoG and Myh2 in C2C12 cultures after 24 h incubation with DM, or DM containing 100 mM myo-inositol. ***=*p*<0.001 for significantly different expression compared to DM cultures. E, Micrographs of H&E stained C2C12 myoblast cultures after 72 h incubation with DM and 24 h treatment with 10 µM ebselen, 5 mM LiCl or 50 µM L-690, 330. F, Average myotube diameter in the treated cultures. *=*p*<0.05 for significantly increased compared to untreated. G, Myotube diameter distribution in the treated cultures. *=*p*<0.05 for significantly increased compared to untreated cultures. H, Micrographs of H&E stained C2C12 myoblast cultures after 72 h incubation with DM and 24 h treatment with 1.25 µM BIO (a GSK-3β inhibitor) or 5 mM LiCl. I, Myotube diameter distribution in the treated cultures. *=*p*<0.05, **=*p*<0.01 and ***=*p*<0.001 for significant difference compared to the untreated control. J, Average myotube diameter in the treated cultures. **=*p*<0.01 and ***=*p*<0.001 for significantly different average diameter compared to untreated.


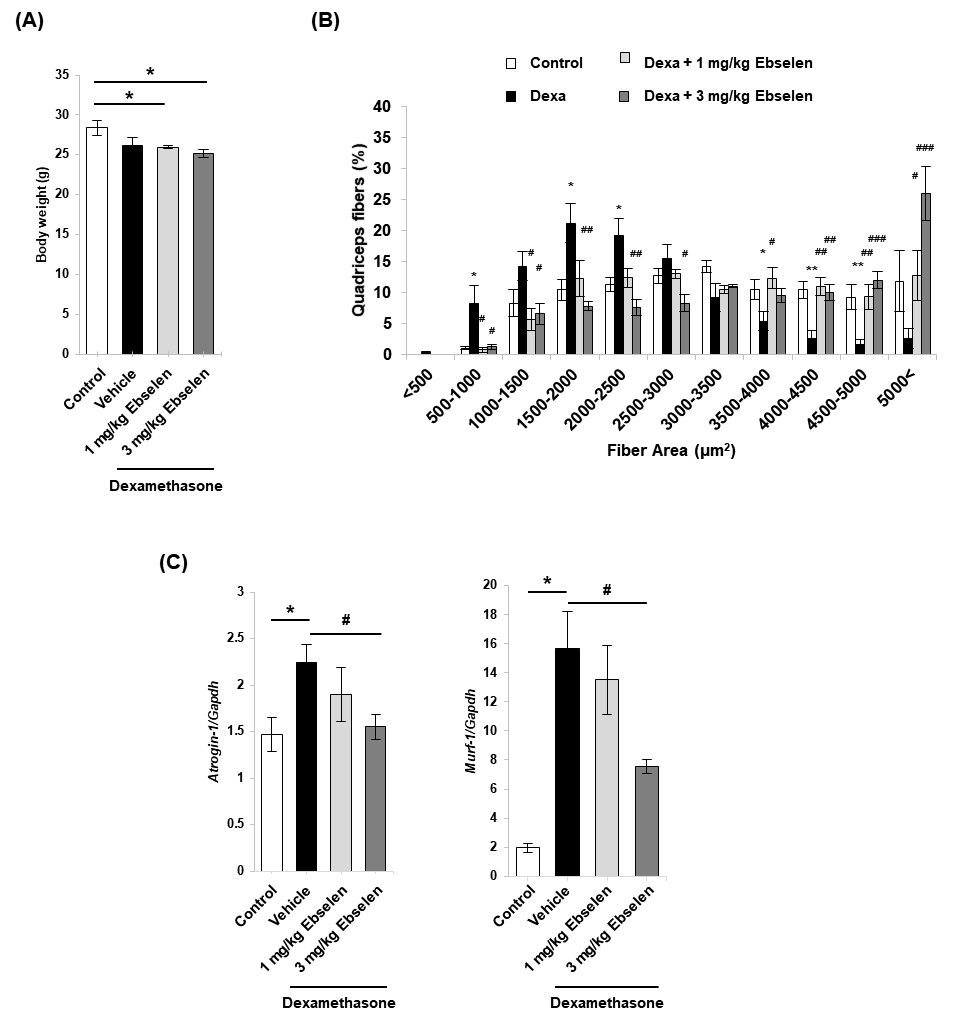


**Supporting Figure 6.** A, Body weight in the dexamethasone treated mice (*=p<0.05 compared to vehicle-treated mice). B, Fiber cross sectional area distribution in the quadriceps muscle. *=p<0.05 and **=p<0.01 for significant difference compared to untreated; #=p<0.05, ##=p<0.01 and ###=p<0.001 for significant difference compared to dexamethasone plus vehicle. C, qPCR analysis of atrogin-1 and MuRF-1 expression in the quadriceps muscle (*=p<0.05 compared to control and #= p<0.05 compared to dexamethasone plus vehicle).


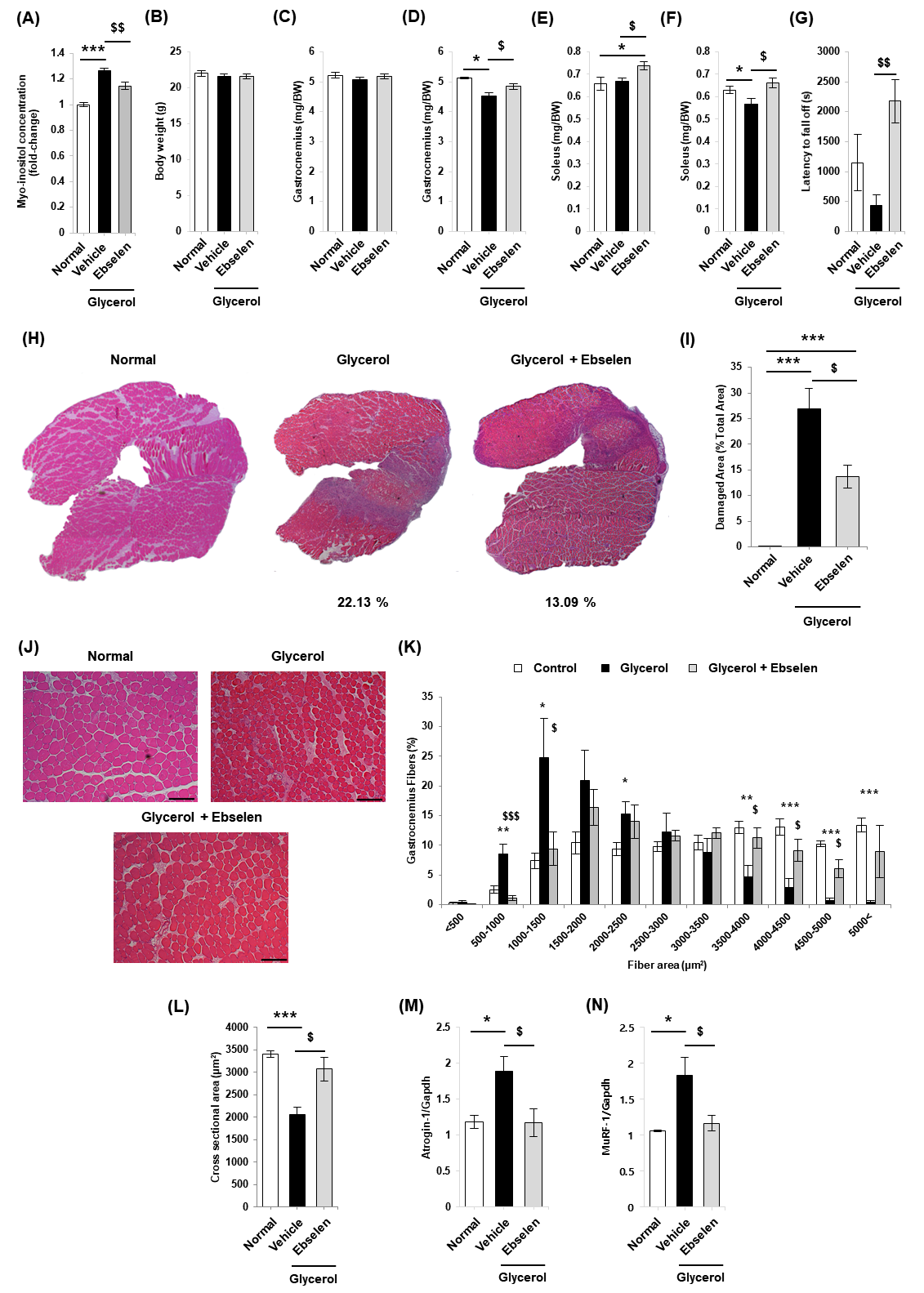


**Supporting Figure 7.** Effect of ebselen on glycerol-impaired skeletal muscle regeneration. A, Myo-inositol level in gastrocnemius muscle of the treated mice. ***=*p*<0.001 for increased myo-inositol compared to untreated mice. ^$$^=*p*<0.01 for decreased myo-inositol compared to vehicle treatment with glycerol injury. B, Body weight in untreated mice (designated as ‘Normal’), mice with glycerol-induced muscle injury, or mice with glycerol-induced muscle injury and ebselen treatment. C, Gastrocnemius muscle mass in normal mice and mice treated with vehicle or ebselen. D, Gastrocnemius muscle mass in the glycerol-injured muscle. **=*p*<0.01 for reduced mass compared to untreated mice. E, Soleus muscle mass in the uninjured muscle of mice treated with vehicle and mice treated with vehicle or ebselen. *=*p*<0.05 for increased mass compared to untreated mice. ^$^=*p*<0.05 for increased mass compared to vehicle treatment. F, Soleus muscle mass in the glycerol-injured muscle. **=*p*<0.01 for reduced mass compared to untreated mice. ^$$^=*p*<0.01 for increased mass compared to vehicle treatment with glycerol injury. G, Latency to fall off in the rotarod system. ^$^=*p*<0.05 for increased latency time compared to vehicle treatment with glycerol injury. H, Representative H&E stained gastrocnemius muscle sections. The numbers beneath each section show the damaged area (%). I, Overall damaged area in the gastrocnemius muscle of the treated mice. ***=*p*<0.001 for increased damaged area compared to normal untreated mice. ^$^=*p*<0.05 for reduced damaged area compared to vehicle plus glycerol injection. J, Representative H&E stained gastrocnemius muscle. Scale bar=200 µm. K, Average cross sectional area in the gastrocnemius muscle. ^***^=*p*<0.001 for decreased average cross sectional area compared to untreated mice. ^$^=*p*<0.05 for increased average cross sectional area compared to mice treated with glycerol plus vehicle. L, Fiber cross sectional area distribution in the gastrocnemius muscle. ^*^=*p*<0.05, ^**^=*p*<0.01 and ^***^=*p*<0.01 for significant difference compared to normal, uninjured; ^$^=*p*<0.05 and ^$$$^=*p*<0.001 for significant difference compared to glycerol plus vehicle. M-N, qPCR analysis of atrogin-1 and MuRF-1 expression in the gastrocnemius muscle. *=*p*<0.05 for increased expression compared to untreated mice. ^$$^=*p*<0.01 for decreased expression compared to vehicle treatment with glycerol injury.

**References**

1. Mosmann, T., *Rapid colorimetric assay for cellular growth and survival: application to proliferation and cytotoxicity assays.* J Immunol Methods, 1983. **65**(1-2): p. 55-63.

2. Sim, W.C., et al., *Activation of SIRT1 by L-serine increases fatty acid oxidation and reverses insulin resistance in C2C12 myotubes.* Cell Biol Toxicol, 2019. **35**(5): p. 457-470.

3. Koguchi, T., et al., *Regulation of myo-inositol biosynthesis by p53-ISYNA1 pathway.* Int J Oncol, 2016. **48**(6): p. 2415-24.

4. Miyake, T., J.C. McDermott, and A.O. Gramolini, *A method for the direct identification of differentiating muscle cells by a fluorescent mitochondrial dye.* PLoS One, 2011. **6**(12): p. e28628.

5. Chiu, H.C., et al., *Preventing muscle wasting by osteoporosis drug alendronate in vitro and in myopathy models via sirtuin-3 down-regulation.* J Cachexia Sarcopenia Muscle, 2018. **9**(3): p. 585-602.

6. Vinel, C., et al., *The exerkine apelin reverses age-associated sarcopenia.* Nat Med, 2018. **24**(9): p. 1360-1371.

7. Bonaldo, P. and M. Sandri, *Cellular and molecular mechanisms of muscle atrophy.* Dis Model Mech, 2013. **6**(1): p. 25-39.

8. Fanzani, A., et al., *Molecular and cellular mechanisms of skeletal muscle atrophy: an update.* J Cachexia Sarcopenia Muscle, 2012. **3**(3): p. 163-79.

9. Antoniadou, I., et al., *Ebselen has lithium-like effects on central 5-HT2A receptor function.* Br J Pharmacol, 2018. **175**(13): p. 2599-2610.

10. Mahdy, M.A.A., *Glycerol-induced injury as a new model of muscle regeneration.* Cell Tissue Res, 2018. **374**(2): p. 233-241.

11. Haddad el, B., et al., *Differential effects of ebselen on neutrophil recruitment, chemokine, and inflammatory mediator expression in a rat model of lipopolysaccharide-induced pulmonary inflammation.* J Immunol, 2002. **169**(2): p. 974-82.
